# Craniomandibular System and Postural Balance after 3-Day Dry Immersion

**DOI:** 10.1371/journal.pone.0150052

**Published:** 2016-02-25

**Authors:** Loïc Treffel, Liubov Dmitrieva, Guillemette Gauquelin-Koch, Marc-Antoine Custaud, Stéphane Blanc, Claude Gharib, Catherine Millet

**Affiliations:** 1 Université Claude Bernard Lyon 1, Lyon, France; 2 Université de Strasbourg, Centre National de la Recherche Scientifique, Unité Mixte de Recherche 7178, Strasbourg, France; 3 Institute of Biomedical Problems, Moscow, Russia; 4 Centre National de la Recherche Scientifique, Unité Mixte de Recherche 6214 - Institut National de la Santé et de la Recherche Médicale 1083, Université d’Angers, Angers, France; 5 Centre National d’Etudes Spatiales, Paris, France; 6 Centre de Recherche Clinique, Centre Hospitalo-Universitaire d’Angers, Angers, France; 7 Centre International d’Ostéopathie, Saint-Etienne, France; 8 Service d’Odontologie, Hospices Civils de Lyon, Lyon, France; University of Brescia, ITALY

## Abstract

The objective of the study was to determine the influence of simulated microgravity by exposure to dry immersion on the craniomandibular system. Twelve healthy male volunteers participated in a 3-day dry immersion study. Before and immediately after exposure we measured maximal bite force using piezoresistive sensors. The mechanical properties of the jaw and cervical muscles were evaluated before, during, and after dry immersion using MyotonPRO. Because recent studies reported the effects of jaw motor activity on the postural stability of humans, stabilometric measurements of center of pressure were performed before and after dry immersion in two mandibular positions: rest position without jaw clenching, and intercuspidal position during voluntary teeth clenching. Results revealed no significant changes of maximal bite force after dry immersion. All postural parameters were significantly altered by dry immersion. There were however no significant differences in stabilometric data according to mandibular position. Moreover the masseter tonicity increased immediately after the end of dry immersion period. Dry immersion could be used as a valid model for studying the effects of microgravity on human subjects. However, 3 days appear insufficient in duration to evaluate the effects of weightlessness on maximal bite force. Our research suggests a link between postural disturbance after dry immersion and masseter tonicity.

## Introduction

Exposure to microgravity induces modification of all physiological systems. Because inflight studies of this modification are difficult, it is necessary to use earth simulation. In both cases, inflight or simulation studies, a deconditioning is observed [1]. “Dry” water immersion has been proposed as an analog to space flight [2]. In this model, the subjects are immersed in a horizontal position up to the neck and separated from the water by an elastic waterproof fabric [3]. Some short-term ground-based studies have shown that dry immersion (DI) can induce many of the deconditioning effects of those occurring in space flights, including sensorymotor, cardiovascular, and musculoskeletal deconditioning [4,5].

Concerning the craniomandibular system (CMS), there is very limited data available in humans on the potential impact of microgravity. Thus, further investigations are needed to determine if the CMS is altered after a microgravity exposure. Actually, the craniofacial complex, being directly affected by the fluid shift during spaceflight or simulated microgravity model, may also induce changes in the soft and hard tissues. Moreover, the reduction of external mechanical loading has detrimental effects on skeletal muscle. However, studies on animal raised the possibility that masticatory muscles do not respond to changes in workload in the same manner as limb muscles [6]. Indeed, masticatory muscles are morphologically and physiologically different from limb muscles [7]. After 13 days of space flight, the masticatory muscles of mouse do not undergo atrophy [8]. Most studies on masticatory muscles have focused on the masseter, which produces the most significant forces during jaw-closing. This muscle is divided into a superficial part involved in bite force production and a deep part more involved in the control of mandibular position. Giannakopoulos et al. reported a co-activation of jaw and neck muscles during submaximal teeth clenching [9]. The maximal bite force has been reported to be different according to population or measuring instruments and techniques [10,11]. For example, the maximal bite force (MBF) among populations in the western hemisphere ranged from 600 to 750 N [10]. However, to our knowledge, no clinical study has focused on the impact of microgravity on the maximal bite force in humans. In the elderly, Miura et al. reported that chewing ability in an “independent” group was greater than in the “bedridden” group without any significant differences in the number of missing teeth [12].

On the other hand exposure to microgravity induces alterations in vestibular and somatosensory systems functioning [13], and head movement control [14], which can participate in the deterioration of upright postural stability after space flight or simulation studies [15]. To evaluate postural control capacity, many studies described recording the ground reaction vector, known as center of pressure (COP), during quiet standing tests in force plate measurement system [16,17]. In normal gravitational conditions, some authors have observed the neuromuscular effects of jaw motor activity on human postural sway during balance tests [18–20]. Numerous neural connections between the CMS and the centers involved in postural control have been proposed to explain these effects [21–23]. Recently, Ringhof et al. reported that postural stability was significantly improved by voluntary clenching of the jaw [24]. However, the correlation between dental occlusion and body posture is discussed controversially [25,26].

The purpose of the present study was to determine the effects of 3-day DI on the CMS. It was hypothesized that (1) DI could change the tone of masticatory and neck muscles and consequently the maximal clenching of the teeth; (2) Jaw clenching would improve postural stability after DI.

This was the first DI study used outside of Russia, in comparison to the common model of head-down bed rest (HDBR). This study was part of a larger group of investigations sponsored by the French National Space Agency (CNES).

## Materials and Methods

### Subjects

**Fig 1:**
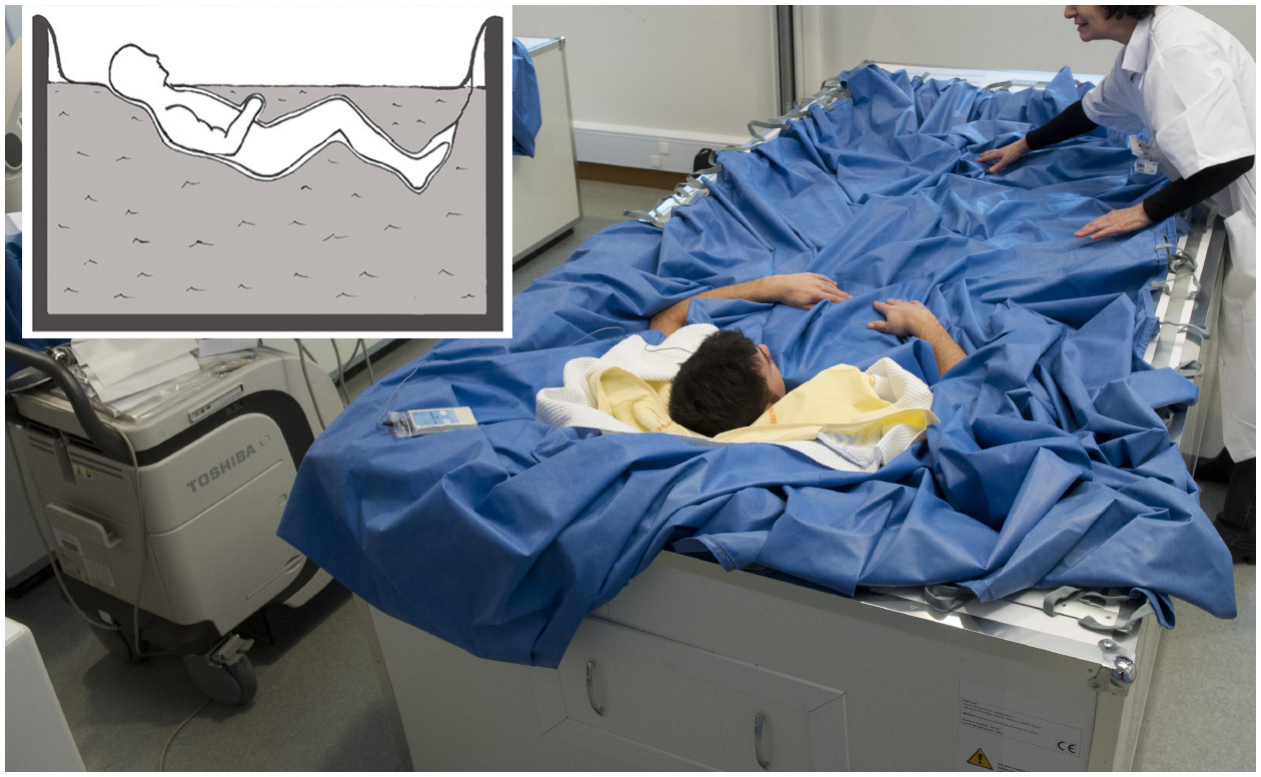
Experimental set-up. The subject is separated from the water by an elastic waterproof fabric.

Twelve healthy male subjects between the ages of 26 and 39 (mean ± SEM one day before DI, age: 31.8 ± 4.1 yr, height: 178.8 ± 5.6 cm; weight: 74.8 ± 5.6 kg; body mass index: 23.6 ± 1.2; aerobic fitness: 38.8 ± 2.9 ml.kg^-1^.min^-1^) participated in this study. All participants were non-smokers, taking no medication or drugs, and received a comprehensive clinical assessment before giving their written informed consent. Study design was established in accordance with the Declaration of Helsinki and was approved by the local ethics committee (CPP Sud-Ouest Outre-Mer I, France) as well as the French Health Authorities. The individual in this manuscript (Fig 1) has given written informed consent (as outlined in PLOS consent form) to publish these case details. The study was organized by the Institute for Space Medicine and Physiology (MEDES-IMPS) in Toulouse, France. The subjects were selected based on a normal clinical investigation consisting of a detailed medical history, physical examination, an electrocardiogram, general blood screening, and urine analyses. Participants were free from muscular or neurological pathologies. All had normal vision, no temporomandibular dysfunction, and full natural dentition (except for third molars) without occlusal disorders.

### Experiment procedure

#### General protocol

This experiment consisted of a 3-day ambulatory control period (BDC-3 to BDC-1), 3 days of dry immersion (DI 1 to DI 3) and 2 days of recovery (R0 to R+1). In the ambulatory and control periods preceding and following DI, all subjects remained active and ambulatory. All were asked not to exercise during the 8 days of the experiment. During DI, the subjects remained immersed in a supine position (Fig 1) in a controlled thermoneutral bath (33 ± 0.5°C) continuously except for a daily 20-minute extraction for toilet procedure and weighting (in bed rest position). The study was conducted in a quiet room at a temperature of ~25°C. During this study, eight different research groups performed their protocols on several physiological systems. The different protocols and hygienic procedures involved a total duration of 4 h 45 min out of immersion between DI 1 and R0. During this period the subjects were maintained in a -6° head-down position. In DI only the head and neck were not entirely immersed in water. The subjects were supervised by medical control and monitored 24 h per day. Room lighting was on between 7:00 AM and 11:00 PM. Each subject had a daily medical examination and several standardized measurements were taken by MEDES personnel. Discomfort and psychological assessments were made via questionnaires. Body temperature was taken twice daily with a tympanic thermometer. Heart rate and arterial blood pressure (systolic, mean and diastolic) were measured every morning by means of an automated sphygmomanometer (Dinamap). The plasma volume was also estimated in the morning (before breakfast) in supine position just before DI (DI 1) and immediately at the end of the DI period, just before standing (R0), using the optimized CO-rebreathing method as described by Caiani et al. [27].

Subjects received three solid meals/day during the study with the requirement to finish all meals. Each menu had similar medium food consistency so as not to change masticatory function. The individual energy intake was calculated by multiplying resting metabolic rate with a physical activity level of 1.6 during pre and post DI and 1.3 during DI. Coffee, tea, alcohol, smoking, and drugs were prohibited throughout the experiment. Only paracetamol was allowed if needed.

#### Maximal bite force

Bite force tests were repeated on the same subjects in the morning three days before DI (BDC-3), then on the first day of recovery (R0), i.e., approximately 45 min after the subjects first stood upright, and then one day later (R+1).

**Fig 2:**
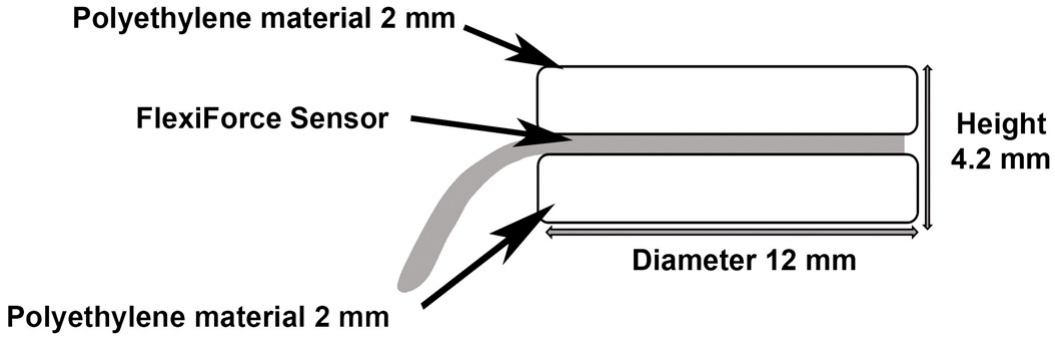
Schematic drawing of custom-made bite force device set up.

MBF was registered in the first molar area after construction of twelve bite force measurement devices (one device per subject). Each device consisted of 2 discs (2.0 mm height x 12 mm diameter) of polyethylene (Duran Scheu-Dental; GmbH, Iserlohn, Germany) separated by a midline disk-shaped pressure sensor (Flexi Force Sensor model B201; Tekscan Inc, Boston, MA, USA). These transducers are thin strips (0.13 mm) with printed piezoresistive sensors of area 9.53 mm^2^ capable of measuring the applied load over the sensing area with a response time of < 5 μs and a linearity error of ± 5% (44, 50). The polyethylene discs were bonded to the sensors using a laminating adhesive (Stamark; 3M, St. Paul, MN, USA). These sensors had long cables covered by a thin vinyl tube, which exited the oral cavity via the oral vestibule at the molar portion. The total device thickness was approximately 4.2 mm (Fig 2). Prior to testing, each individual sensor was calibrated at ambient temperature (20°C) at a loading range of 0–1000 N by laboratory tests with known force values using a material testing machine (Sun 1000; Galdabini, Cardano al Campo, Italy). The sensors were connected to a computer using a sensor connecting device provided by the same manufacturer (Tekscan) and data were collected at intervals of 0.125 s using the Tekscan ELF specific software (converting pressure values into forces). Data were exported to a spreadsheet for storage and further processing.

During testing the volunteers were seated comfortably in an upright position with the Frankfort plane parallel to the floor. The sensor was positioned unilaterally on the mandibular first molar and the subjects were asked to clench as hard as possible for 2–3 sec; according to a standard procedure [28]. Bite force was measured alternately on the right and left sides with adequate rest intervals between each bite to prevent muscle fatigue. Subjects were encouraged verbally to perform maximal clenching with each repetition. Two readings were obtained on each side. From these recordings, three values were used in the analysis: the average on each side and the average of the four readings. The measurement method was explained in detail to all subjects before it was performed. No participant reported pain during or after measurements.

*Method error*: The MBF measurements were taken twice by the same investigator after 1 week using 10 healthy volunteers and the identical procedure and then random errors of the MBF measurement were assessed by Dahlberg’s formula [29]. Using Dahlberg’s formula (√Σd2/2n), Σd2 denotes the sum of the squared differences between pairs of recordings while n denotes the number of duplicate measurements. MBF measurement error was 51.8 N (7.6%) and the coefficient of reliability was 0.91.

#### Mechanical properties of muscles

The mechanical characteristics of the masseter and sternocleidomastoid (SCM) muscles were determined at rest using a hand-held myometer (MyotonPRO; Myoton Ltd, Estonia) during the last day of the baseline data collection period (BDC-1), on days 1 and 3 of the DI period, and after DI on the first day of recovery (R0). This method measures the viscoelastic response of the muscle due to a brief (15 milliseconds) mechanical impulse (force 0.4 N) on the skin surface above the muscle. The mechanical deformation is delivered by the device testing end (d = 3 mm) held perpendicular to the skin surface. An integrated 3-axis digital acceleration sensor recorded the muscle oscillation in the form of an acceleration curve. The properties of the sensor are: amplitude range of ± 8 g in full range; resolution of 11 bits; output data rate and bandwidth 3,200 Hz; sensitivity ± 0.01% due to the temperature change; and operating temperature −10 to +55°C. If a curve failed to meet measurement parameters, an error message warned the experimenter to repeat the trials. The device was used in multiscan mode, where one measurement corresponded to the mean of 5 mechanical taps. The procedures have been reported in the literature [30–32]. These studies have demonstrated the validity and reliability of Myoton measures in limb, trunk, and orofacial musculature. From the oscillation acceleration signal, we investigated three parameters computed in real time by MyotonPRO software: dynamic stiffness, oscillation frequency, and mechanical stress relaxation time. Dynamic stiffness characterizes the resistance of the muscle to the force that changes its shape. This parameter was calculated as follows: Stiffness = *m* × *a*_max_*/* Δ*l*, where *m* is the mass of the testing end of myometer (kg); *a*_max_ is the maximal acceleration of oscillation (m/s^2^); Δ*l* is the deformation depth of the muscle mass [33], with a reliability/precision of 3.9%. Oscillation frequency, which characterizes the muscle tone or the mechanical tension in a relaxed muscle, was as follows: *f* = 1/*T* [Hz], where T denotes the oscillation period in seconds [34] with a precision of 1.1%. Mechanical stress relaxation time is the time for the muscle to restore its initial shape after external force is removed (viscoelastic properties) [35]. It was measured with a reliability of 1.5% (Testing certificate no. 2-034-11 in 09-09-2011).

During the tests, the subject lay in a relaxed supine position. Bilateral measurements are taken on the right and left masseter and SCM muscles in a complete rest position in order to measure relaxed muscle properties. The device-testing end was placed perpendicular to the surface of the main portion of the masseter, 20 mm from the inferior edge of the mandibular angle (gonion), and on SCM muscle at the lower 1/3 of the line connecting sternal notch and mastoid process. Anatomic landmarks were marked with a pen and all the measurements were taken on the same muscle point. All collected values were downloaded from the device to a computer for data analysis.

#### Postural sway measurement

Posturographic tests were repeated three days before DI (BDC-3), then on the first day of recovery (R0), i.e., approximately 30 min after the subjects first stood upright, and one day later (R+1). All static posturographic tests were quantified by recording modifications in the position of the COP on a force platform (Leonardo Mechanograph Ground Reaction Force Plate; Novotec Medical GmbH, Pforzheim, Germany). The platform was composed of two symmetrical force plates with four sensors. Force was detected by the deformation of the sensors proportionally to the applied force (change in electrical resistance). The signal from the eight force sensors was sampled at a frequency of 800 Hz and was analyzed using the Leonardo Mechanography GRFP Research Edition software (version v4.1.19-RES; Novotec Medical). The force platform was positioned in the floor and was adjusted to indicate a mass of zero kg before the subject gets up on it. All volunteers were lightly dressed and did not wear shoes during the tests. They were required to stand stable on the 66×66 cm platform in a comfortable upright orthostatic position with both arms hanging free beside the trunk. They were asked to breathe normally and look straight ahead fixating their gaze on a precise point at eye level on a wall approximately 2.5 m away. The anteroposterior (AP) and mediolateral (ML) positions were determined by the use of marks on the platform corresponding to an inter-malleolar distance of ~15 cm. For each subject, two different mandibular positions were analyzed (1) dental intercuspidal position (ICP), obtained by asking the subject to close the mouth and moderately clench the teeth with a “medium” force range for 30s; (2) rest position (RP) without contacts between the dental arches. All tests were recorded in eyes open (EO) and eyes closed (EC) conditions in randomized order. Data recording started once the subject was stable using an audible tone. A double-audible tone indicated the end of the trial. Each trial lasted 30 s without modification of the position of feet on the platform between tests, and in quiet conditions. Rest periods of 30–60 s were provided between trials. Among the traditional parameters collected, this article reports the results of total path length, sway area, mean velocity, and the length function of surface (LFS) of the COP displacements.

The total COP path length was quantified from the COP displacements in the anterior-posterior (AP_d_) and mediolateral (ML_d_) directions. Total COP path length = [(Displacement in AP_d_)^2^ + (Displacement in ML_d_)^2^]^0.5^ [17]. This parameter describes the direction and extent of postural sway [19]. The calculation of the sway area provided by the software considered the area of the confidence ellipse that encloses 95% of the most concentric points of the COP path during the 30 s test. This conventional procedure automatically eliminated 5% of the extreme points in order to delete values which could be induced by quasi voluntary movements not indicative of the amount of postural sway [36]. The sway area reflects the precision of postural control, the smaller the COP area the better the stability [37]. The velocity (i.e., COP path length divided by trial duration) has been suggested to represent the amount of activity required to maintain stability, the smaller the COP velocity the better the postural control [37]. The length function of surface (LFS) was also calculated with the following equation: LFS = sway path/396 x exp^(0.0008×area)^ [36]. It provides information about the precision of postural control and the energy used by the participant to stand steady [36].

Before data collection began, all subjects were familiarized with the tools and the test structure. Maximal bite force assessment, Myoton measurements, and postural stability were performed by a single trained investigator at the same time of day to avoid any potential circadian effects.

### Statistical analysis

The present study was designed to examine the effects of 3 days of DI on bite force, mechanical properties of muscles, and postural stability.

Analyses were performed using the StatView software version 5.0.1 (SAS Institute, Cary, NC, USA). The effects of DI on monitoring daily variables were tested by repeated measures analysis of variance (ANOVA). For muscle and bite data, a two-way repeated measures ANOVA was performed with time period and side (left and right) as factors. ANOVA for repeated measures was also used to evaluate changes in the postural stability over three repeated measures: 1) time period (3 levels: BDC-3, R0, R+1), 2) eye condition (2 levels: eyes open and eyes closed), and 3) mandibular position (2 levels: dental intercuspidal position and rest position). Where overall significance occurred, post hoc testing was conducted using Fisher’s protected least significant difference. All data are presented as means ± SE; the 5% level (*p* < 0.05) was chosen as statistically significant.

Because there were no significant differences between sides of the body with muscle properties, right and left side values were averaged for analyses.

## Results

### Monitoring daily variables

Table 1 shows pre- and post-DI monitoring daily variables. We observed a significant decrease in plasma volume versus baseline of -17 ± 5% on day R0 (*p* < 0.001) and in weight. Mean weight loss after DI was 1.2 ± 0.6 kg. Resting heart rate (HR), systolic blood pressure (SBP), diastolic blood pressure (DBP), and temperature remained unchanged following DI.

**Table 1:** Body temperature, body weight, plasma volume, systolic and diastolic pressure, and heart rate before and after dry immersion.

	Pre-DI (DI 1)	Post-DI (R0)	*p* value
	(n = 12)	(n = 12)	
**Morning temperature, °C**	36.5 ± 0.2	36.4 ± 0.1	0.317
**Weight, kg**	74.5 ± 5.9	73.3 ± 5.6 ***	< 0.0001
**Plasma volume, ml**	3727.3 ± 109.9	3095.6 ± 86.7 ***	< 0.0001
**Systolic blood pressure, mm Hg**	119.7 ± 7.2	120.9 ± 8.6	0.693
**Diastolic blood pressure, mm Hg**	65.5 ± 4.0	68.2 ± 3.7	0.077
**Heart rate, beats/min**	54.8 ± 5.7	58.1 ± 8.4	0.202

Days are indicated as follows: dry immersion (DI) day 1 (day DI 1), and recovery day (R0). Values are means ± standard error.

***p < 0.001 compared with DI 1 value.

### Maximal bite force

Bite force data at the different periods are listed in Table 2. Statistical tests revealed no significant changes in MBF between the periods. No significant right-left differences were observed for the measurements of MBF (*p* > 0.05).

**Table 2:** Maximal molar bite force before and after dry immersion.

	BDC-3	R0	R+1	*p* value
	(n = 12)	(n = 12)	(n = 12)	
**MBF right (R)**	632.5 ± 42.2	702.7 ± 53.0	673.6 ± 49.3	0.244
**MBF left (L)**	666.1 ± 39.9	654.3 ± 42.5	688.4 ± 38.7	0.418
**Mean MBF (R/L)**	649.3 ± 38.4	679.3 ± 42.8	680.9 ± 38.6	0.484

Maximal molar bite force (MBF) on the right (R) and left (L) sides. Days are indicated as follows: control period (BDC-3), the first day of recovery (R0), the second day of recovery period (R+1). Units are N; Values are means ± standard error. All comparisons were not statistically significant.

### Mechanical properties of muscles

Masseter and sternocleidomastoid muscle stiffness, frequency and relaxation time data are shown in Table 3. For technical problems, the data of 3 subjects have not been analyzed. Since all remaining data showed no difference between the right and left sides (all *p* > 0.05), the results were pooled.

**Table 3:** Combined (left and right) changes in jaw and cervical muscles stiffness, frequency and relaxation time before, during and after dry immersion.

	BDC-1	DI 1	DI 3	R0	*p* value
Parameter	(n = 9)	(n = 9)	(n = 9)	(n = 9)	
**Masseters**					
Stiffness (N. m^-1^)	334.1 ± 8.0	329.8 ± 10.3	323.2 ± 15.5	367.9 ± 21.3 *	0.028
Frequency (Hz)	16.5 ± 0.3	16.2 ± 0.3	16.2 ± 0.4	17.8 ± 0.7 *	0.003
Relaxation time (ms)	16.7 ± 0.4	16.7 ± 0.4	17.3± 0.8	15.2 ± 0.7 **	0.011
**Sternocleidomastoid**					
Stiffness (N. m^-1^)	211.0 ± 17.7	190.3 ± 8.9	199.8 ± 12.0	197.8 ± 10.5	0.485
Frequency (Hz)	13.7 ± 0.5	13.1 ± 0.3	13.4 ± 0.3	13.4 ± 0.3	0.478
Relaxation time (ms)	23.2 ± 0.8	24.3 ± 0.7	23.5 ± 0.8	23.4 ± 0.9	0.663

Days are indicated as follows: the last day of control period (BDC-1), the first day (DI 1) and third day (DI 3) of dry immersion, and the first day of recovery period (R0). Values are means ± standard error.

*p < 0.05;

**p < 0.01 compared with BDC-1 value.

None of the three parameters of sternocleidomastoid muscles were changed during the experiment. Concerning masseter data, there was a significant period effect for stiffness (*F*_*(3*,*24)*_ = *3*.*607*, *p* = 0.028), frequency (*F*_*(3*,*24)*_ = *5*.*932*, *p* = 0.003), and relaxation time (*F*_*(3*,*24)*_ = *4*.*584*, *p* = 0.011). Post-hoc analysis shows that stiffness and frequency (tone) increased immediately after DI on R0 (*p* = 0.04 and *p* = 0.02, respectively), and relaxation time was significantly decreased on R0 (*p* = 0.002).

### Postural sway parameters

**Fig 3:**
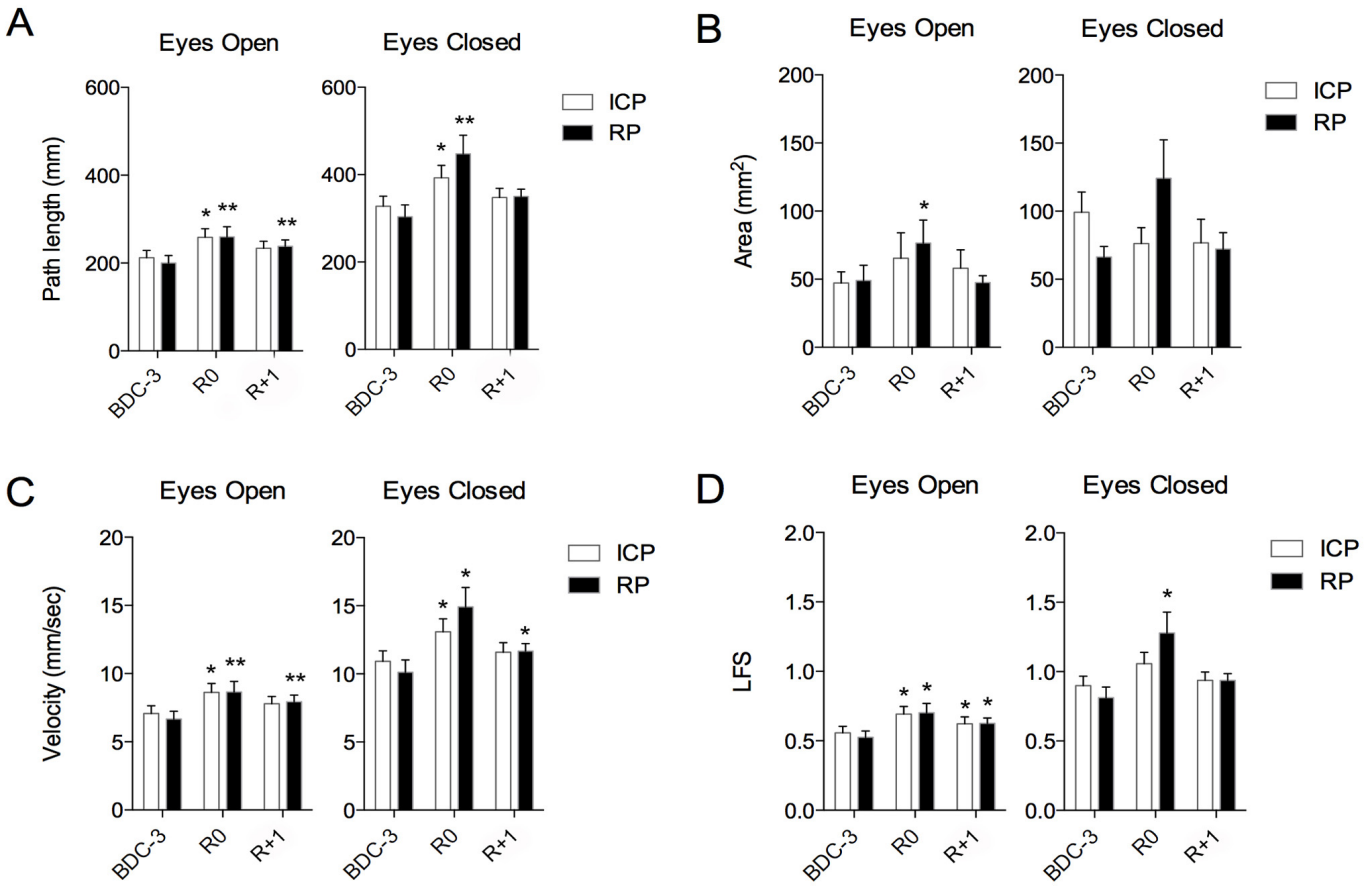
Mean ± SE values of postural parameters. Mean and standard error values of center of pressure (COP) path length (A), area (B), velocity (C), and length function of surface (LFS) (D) during control period (BDC-3), the first day (R0) and the second day of recovery period (R+1) for the different test conditions: dental intercuspidal position (ICP) (white bars) and mandibular rest position (RP) (black bars), eyes open and eyes closed. * and ** significant differences compared with BDC-3 value. (Respectively *p* < 0.05 and *p* < 0.01; ANOVA for repeated measures).

There was a strong period effect (after *vs* before DI) on all posturographic parameters (Fig 3), path length, velocity, LFS (all *p* < 0.0001), and area (*p* = 0.043). Post-hoc analysis shows that the subjects were more unstable on getting up (data greater for R0 and R+1 than for BDC-3), and degradation of performance is most important at R0 (S1 Table). There was also a strong vision effect on all posturographic parameters: path length, velocity, LFS (all *p* < 0.0001), and area (*p* = 0.007). Thus, regardless of the period, vision suppression degrades postural performances. Regarding COP path length and LFS, the results in EC were not significantly higher at R1 compared with BDC-3 while results in EO were significantly higher at R1 compared with BDC-3 (*p* < 0.05). The recovery of pre-DI performances seems slower in EO condition.

About dental occlusion, regardless of the eye condition, there was a tendency for a better postural stability on getting up at R0 for dental ICP than for mandibular RP, but this tendency did not reach statistical significance. Regarding COP path length, and although the data were slightly larger in mandibular RP than for dental ICP at R0 and R+1, they were not statistically significant (*p* = 0.96 and *p* = 0.58 respectively in EO condition, and *p* = 0.16 and *p* = 0.89 respectively in EC condition). Similarly for the COP area and velocity, and although the period effect was reduced in dental ICP compared with mandibular RP, these differences were not statistically significant.

## Discussion

To our knowledge, the present study was the first to examine the CMS in DI, and more generally in a microgravity simulated study. The main results from this study were: 1) 3 days of DI were not enough to change the maximal bite force; 2) masseter stiffness and frequency were increased on getting up after DI; 3) we have demonstrated changes in postural balance following short-duration DI. However, “medium” jaw clenching had no effect on postural sway.

### Monitoring daily variables

In the present study, in spite of the short duration of DI, plasma volume was significantly reduced by 17.0 ± 5%. This reduction is comparable to that observed in six astronauts during their first day in flight (-17%) by Alfrey et al. [38] and confirms previous HDBR data [39].

### Maximal bite force

Concerning the stable value of MBF, it could be explained by a normal activity of masticatory muscles during DI. Moreover, the head was almost entirely out of water, which reduced the impact of simulated microgravity on the cervical and craniomandibular regions. In addition, the subjects needed to do a flexion of the head in order to facilitate the oropharyngeal phase of swallowing after chewing food. This head-flexed position widens the vallecular space to prevent the bolus from entering the airway, and puts the epiglottis in a more protective position [40]. Furthermore, food consistency during DI was similar to the standard conditions on earth (solid diet), whereas foods generally have a softer consistency during space flights. In fact, a clear relationship between muscular activity and food properties has been reported in humans. Food texture modifies masticatory forces [41]. Foods with different consistencies have been used to change the loading to rat jaw muscles to study the adaptation capacity in the CMS [42,43]. All these animal studies lasted several weeks and demonstrated that a decrease in the amount of mastication and thus occlusal forces affects all structures, including bone and muscles. As was demonstrated in a recent study by Philippou et al. [8], the superficial masseter fiber size of mouse was not affected by microgravity after 2 weeks on a hard diet aboard the Shuttle Atlantis. In contrast, the superficial masseter fiber size decreased significantly after 2 weeks on a liquid diet. The authors concluded that the lack of mass loss in masseter muscles could be a protective effect, at least for the short period of 2 weeks of space flight, by chewing-induced loading of these muscles. Thus, the evaluation of weightlessness on MBF in humans requires experiences of longer duration and chewing foods with consistencies similar to those used by astronauts in flight.

Analysis of results showed that subjects had approximately equal MBF on the right and left side, which is in agreement with previous studies [11,44]. The bite force level is related to biological factors such as sex, dental status, craniomandibular morphology, muscle strength, and neuromuscular feedback mechanisms [45,46]. Additionally, bite force varies in the different regions of the oral cavity and is largest in the first molar area [47]. However, the accuracy of bite force recording is also influenced by the occlusal-force measuring device used. Custom-made devices with strain gauge are sometimes used but their significant thickness leads to a greater distance between antagonistic teeth. The results may therefore not be reliable [45]. Thus, in the current study, piezoresistive sensors were used because of their small thickness [40]. The interocclusal distance promoted between molars by the device was approximately 4 mm. Moreover it has been demonstrated that these sensors are able to record intraoral force levels with sufficient clinical accuracy and precision [46].

### Postural sway

In agreement with previous HDBR studies [16,48], the results of the present study demonstrated a significant alteration of static posturographic parameters immediately after DI at R0 regardless of the eye condition. We observed a significant impairment of balance control after DI, as in bed rest studies where equilibrium scores are lower just after bed rest [49]. For example, concerning the path length, we found 21% increase, as well as in Viguier et al. [48] with 31% increase after 60 days in HDBR. In the same way we found an increase in velocity parameter after DI of 25% on average, in comparison to Muir et al. [16], which noted a velocity rise of 192.7%. The comparison between these two studies is difficult. Even if our results were significant after DI, these differences would be explained by a comparison to a long period of bed rest, which impaired more particularly the muscular mass. Indeed balance control depends largely on tonic muscle activity. Here the severe loss of postural muscle tone is one of the early manifestations of exposure to dry immersion [5] and is certainly involved in the postural performance. Neuromotor changes after DI lead to disturbances in coordination, which result in decreased accuracy in muscle effort and a decreased performance in postural stability [3]. Maintaining postural balance on Earth is a complex control task for the central nervous system, which must integrate several types of afferent inputs: exteroceptive (skin sensitiveness of feet), proprioceptive (especially from the cervical, hip, ankle, and knee joints), vestibular (semicircular canals and otoliths), and visual (movement of the surrounding environment) [13,50]. Exposure to microgravity induces sensorimotor changes which can participate in the deterioration of upright postural stability after returning to normogravity conditions [13–15]. It seems important to state that DI is an environment that influences proprioceptive feedback but probably not vestibular activity and visual feedback. This may be a limitation to this reported study. In contrast, numerous space flight studies have demonstrated otolith deconditioning [51,52]. In weightlessness, there is an absence of gravitationally based otolith stimuli to the central nervous system, which creates a sensory conflict that appears to be the cause of the disorientation and motion sickness experienced by many astronauts during the first few days in flight [51]. Therefore, postural control is certainly more disrupted after exposure to real microgravity as compared with DI. Indeed posturography tests performed daily after return of the Space Shuttle showed significant impairments in equilibrium control on landing day [13,51]. In the present study, higher LFS was systematically observed with suppression of vision which means more energy expenditure to maintain balance in EC condition.

Although sway parameters were more altered after DI in RP condition, voluntary teeth clenching did not significantly influence the stabilometric data compared with a habitual non-biting condition. So the influence of dental occlusion on static postural performance can not be confirmed in this study. This agrees with a meta-analysis reported by Perinetti et al. [53] and with several experimental results [25,26] in normal gravitational conditions. However, some authors reported a functional interdependence between the dental occlusion and posture [18,19]. For example Cuccia [50] showed a correlation between teeth clenching and an increase in plantar surface area (with a decrease in plantar loading) on a baropodometric platform; however the author investigated subjects during walking and not in static positions. This suggests that it could be relevant to investigate dental occlusion during dynamic movements in further studies. Recently published results showing that voluntary teeth clenching significantly affected neuromuscular co-contraction patterns and improved postural stability in upright stance [24,54]. The authors assumed that teeth clenching facilitated human motor excitability [55] and resulted in nonreciprocal facilitation of ankle extensor and flexor muscles, and attenuated reciprocal Ia inhibition of the pretibial muscles and the soleus muscle [56]. In view of the literature, it appears that controversial correlation between the body posture and the CMS may be partly related to the magnitude of bite force. In most publications, information of the jaw positions and bite-forces are insufficient. The force level is generally not examined and commonly left to the subjects’ self-assessment. It could well be that differences in the bite force between the different dental conditions are one of the reason for the changes/or not changes in the postural performance. For this reason, the development of studies with valid measurement devices of teeth clenching appears to be relevant to determine the association between the dental occlusion and the postural performance. In addition, the majority of the studies on dental occlusion and body posture revolve around the relationship between malocclusions and anomalies in the position of the craniocervical tract. An alteration of dental occlusion may have an impact on spinal column alignment and can reasonably influence the body muscular equilibrium. This is why the subjects of the present study were selected without dental and temporomandibular disorders. Finally, for all the reasons mentioned above, it is suggested that the evaluation of weightlessness on postural performances using force platforms must be performed without contact between opposing teeth.

### Mechanical properties of muscles

About masticatory and neck muscles, the study showed a significantly higher stiffness and frequency of the masseters at recovery (R0), which is the moment when postural balance is the most disturbed. Although the exact mechanism is unclear and discussed, one possible explanation is the connection between the vestibular system and the excitatory of the masseter muscles, in reference to Deriu et al. [57], and Tolu and Pugliatti [58]. These authors showed the relation between the two systems, validating the existence of a vestibulomasseteric reflex. The authors have shown a lot of neurological connections between the trigeminal and the vestibular nuclei in animals and humans. Moreover morphological studies have shown reciprocal connections between the vestibular nuclear complex and spinal trigeminal nuclei in rats [59,60]. To be more precise, Deriu et al. [57] describes two sets of functional connections between the vestibular system and the masseter motoneurons. There is a short latency pathway that may allow vestibular inputs rapid access to jaw muscle control, and an indirect pathway that may be essential to postural control of masseters by stabilizing the jaw during head motions [57]. The authors conclude that trigeminal motoneurons are tonically and bilaterally excited and macular inputs exert a bilateral asymmetrical control on jaw muscles in relation to angular acceleration of the head in spatial planes [57,61]. In anatomical study in rats, labelled neurons were observed bilaterally in the inferior vestibular nucleus with an ipsilateral predominance [59]. Studies were confirmed in humans the vestibulo-trigeminal relationship described in the animal model, and then the support for the bilateral polysynaptic vestibulo-trigeminal pathways [57,62].

In a certain way, we think that a perturbation of postural balance, here by DI conditions, could activate the vestibulomasseteric reflex by the intermediary of vestibular perturbations in order to have more spatial information regarding the head position in space, particularly with eyes closed. It could lead to an increase as a rebound effect, in the masseteric tonic activity at recovery, particularly in the first moments post tilt where the postural control is the worst, with the still aim to keep the up-right position more stable. An electromyographic monitoring of resting masseter muscle activity could be used in future studies to confirm this supposition. Moreover it could be interesting to expand the study to others masticatory muscles such as temporal, that, with masseter are the two main muscles of mastication. Future research should also measure the response of the posterior cervical muscles (e.g. splenius capitis and semispinalis capitis) that are considered to have an important role for eye-neck (head) stabilization during standing.

In addition, psychological stress could have contributed to produce the differences observed at R0. There is a concentration of all the measurements at R0, particularly the Tilt test that could stress the subjects at recovery. Moreover the subjects have very bad nights with a global discomfort during all DI of 40 ± 6.6 (Mean ± SD) on a scale from 0 to 100, which could lead to an asthenia. Indeed the integration of the masticatory system with the brainstem center via the sensorimotor system may explain the sensitivity of the masticatory system to different descending stimuli (stress, anxiety, asthenia, etc.).

## Conclusion

This study reveals that there is no evidence of masticatory muscles and MBF dysfunctions during 3 days of DI. Therefore, it would be interesting to study the CMS over a longer period of simulated microgravity. However, the DI model, even during just 3 days, appears to be a very accurate and rapid model for simulating effects of microgravity on postural sway. Our results suggest a link between postural disturbance after DI and masseter tonicity. Posturographic measurements of the COP were not affected by dental occlusion. However it should be mentioned that postural sway parameters were measured in healthy subjects without dental or temporomandibular disorders. We would like to point out to postural researchers that dental occlusion disorder could disturb the postural balance. Ideally, jaw clenching should be taken into account as a condition, like EC and EO, in postural studies.

## Supporting Information

S1 Table Postural sway parameters before, during and after dry immersion. (PDF)
(PDF)

## References

[1] Schneider S, Convertino V. Physiological systems and their responses to conditions of microgravity and bed rest ACSM’s Advanced Exercise Physiology. Philadelphia, PA: Lippincott Williams & Wilkins: Tipton CM; 2006.

[2] Shulzhenko E, Vil-Vilyams I. The possibility to maintain a long term water immersion by using the method of “dry immersion”. Kosm Biol Aviakosm Med. 1976;10: 82–84. 1263423

[3] Navasiolava NM, de Germain V, Levrard T, Larina IM, Kozlovskaya IB, Diquet B, et al Skin vascular resistance in the standing position increases significantly after 7 days of dry immersion. Auton Neurosci Basic Clin. 2011;160: 64–68. 10.1016/j.autneu.2010.10.003 21071283

[4] Iwase S, Sugiyama Y, Miwa C, Kamiya A, Mano T, Ohira Y, et al Effects of three days of dry immersion on muscle sympathetic nerve activity and arterial blood pressure in humans. J Auton Nerv Syst. 2000;79: 156–164. 10699647 10.1016/s0165-1838(99)00076-4

[5] Kozlovskaia IB. [Fundamental and applied objectives of investigations in immersion]. Aviakosmicheskaia Ekol Meditsina Aerosp Environ Med. 2008;42: 3–7. 19192530

[6] Aare S, Ochala J, Norman HS, Radell P, Eriksson LI, Göransson H, et al Mechanisms underlying the sparing of masticatory versus limb muscle function in an experimental critical illness model. Physiol Genomics. 2011;43: 1334–1350. 10.1152/physiolgenomics.00116.2011 22010006

[7] Monemi M, Kadi F, Liu JX, Thornell LE, Eriksson PO. Adverse changes in fibre type and myosin heavy chain compositions of human jaw muscle vs. limb muscle during ageing. Acta Physiol Scand. 1999;167: 339–345. 10632637 10.1046/j.1365-201x.1999.00624.x

[8] Philippou A, Minozzo FC, Spinazzola JM, Smith LR, Lei H, Rassier DE, et al Masticatory muscles of mouse do not undergo atrophy in space. FASEB J Off Publ Fed Am Soc Exp Biol. 2015;29: 2769–2779. 10.1096/fj.14-267336 PMC4478801 25795455

[9] Giannakopoulos NN, Schindler HJ, Rammelsberg P, Eberhard L, Schmitter M, Hellmann D. Co-activation of jaw and neck muscles during submaximum clenching in the supine position. Arch Oral Biol. 2013;58: 1751–1760. 10.1016/j.archoralbio.2013.09.002 24200301

[10] Al-Omiri MK, Sghaireen MG, Alhijawi MM, Alzoubi IA, Lynch CD, Lynch E. Maximum bite force following unilateral implant-supported prosthetic treatment: within-subject comparison to opposite dentate side. J Oral Rehabil. 2014;41: 624–629. 10.1111/joor.12174 24720815

[11] Arima T, Takeuchi T, Honda K, Tomonaga A, Tanosoto T, Ohata N, et al Effects of interocclusal distance on bite force and masseter EMG in healthy participants. J Oral Rehabil. 2013;40: 900–908. 10.1111/joor.12097 24033381

[12] Miura H, Araki Y, Umenai T. Chewing activity and activities of daily living in the elderly. J Oral Rehabil. 1997;24: 457–460. 9219992 10.1046/j.1365-2842.1997.00530.x

[13] Layne CS, Mulavara AP, McDonald PV, Pruett CJ, Kozlovskaya IB, Bloomberg JJ. Effect of long-duration spaceflight on postural control during self-generated perturbations. J Appl Physiol Bethesda Md 1985. 2001;90: 997–1006. 10.1152/jappl.2001.90.3.997 11181611

[14] Bloomberg JJ, Peters BT, Smith SL, Huebner WP, Reschke MF. Locomotor head-trunk coordination strategies following space flight. J Vestib Res Equilib Orientat. 1997;7: 161–177. 9178222

[15] Souvestre PA, Landrock CK, Blaber AP. Reducing incapacitating symptoms during space flight: is postural deficiency syndrome an applicable model? Hippokratia. 2008;12 Suppl 1: 41–48. 19048092 PMC2577399

[16] Muir J, Judex S, Qin Y-X, Rubin C. Postural instability caused by extended bed rest is alleviated by brief daily exposure to low magnitude mechanical signals. Gait Posture. 2011;33: 429–435. 10.1016/j.gaitpost.2010.12.019 21273076 PMC3050431

[17] Onambele GL, Narici MV, Maganaris CN. Calf muscle-tendon properties and postural balance in old age. J Appl Physiol Bethesda Md 1985. 2006;100: 2048–2056. 10.1152/japplphysiol.01442.2005 16455811

[18] Baldini A, Nota A, Cravino G, Cioffi C, Rinaldi A, Cozza P. Influence of vision and dental occlusion on body posture in pilots. Aviat Space Environ Med. 2013;84: 823–827. 23926657 10.3357/asem.3541.2013

[19] Ringhof S, Stein T, Potthast W, Schindler H-J, Hellmann D. Force-controlled biting alters postural control in bipedal and unipedal stance. J Oral Rehabil. 2015;42: 173–184. 10.1111/joor.12247 25354425

[20] Sforza C, Tartaglia GM, Solimene U, Morgun V, Kaspranskiy RR, Ferrario VF. Occlusion, sternocleidomastoid muscle activity, and body sway: a pilot study in male astronauts. Cranio J Craniomandib Pract. 2006;24: 43–49. 10.1179/crn.2006.008 16541845

[21] Bracco P, Deregibus A, Piscetta R. Effects of different jaw relations on postural stability in human subjects. Neurosci Lett. 2004;356: 228–230. 10.1016/j.neulet.2003.11.055 15036636

[22] Gangloff P, Perrin PP. Unilateral trigeminal anaesthesia modifies postural control in human subjects. Neurosci Lett. 2002;330: 179–182. 12231441 10.1016/s0304-3940(02)00779-6

[23] Marfurt CF, Rajchert DM. Trigeminal primary afferent projections to “non-trigeminal” areas of the rat central nervous system. J Comp Neurol. 1991;303: 489–511. 10.1002/cne.903030313 1706735

[24] Ringhof S, Leibold T, Hellmann D, Stein T. Postural stability and the influence of concurrent muscle activation—Beneficial effects of jaw and fist clenching. Gait Posture. 2015; 10.1016/j.gaitpost.2015.09.002 26385200

[25] Marini I, Alessandri Bonetti G, Bortolotti F, Bartolucci ML, Gatto MR, Michelotti A. Effects of experimental insoles on body posture, mandibular kinematics and masticatory muscles activity. A pilot study in healthy volunteers. J Electromyogr Kinesiol Off J Int Soc Electrophysiol Kinesiol. 2015;25: 531–539. 10.1016/j.jelekin.2015.02.001 25707996

[26] Perinetti G, Marsi L, Castaldo A, Contardo L. Is postural platform suited to study correlations between the masticatory system and body posture? A study of repeatability and a meta-analysis of reported variations. Prog Orthod. 2012;13: 273–280. 10.1016/j.pio.2011.12.003 23260538

[27] Caiani EG, Massabuau P, Weinert L, Vaïda P, Lang RM. Effects of 5 days of head-down bed rest, with and without short-arm centrifugation as countermeasure, on cardiac function in males (BR-AG1 study). J Appl Physiol Bethesda Md 1985. 2014;117: 624–632. 10.1152/japplphysiol.00122.2014 25080927

[28] Bakke M, Michler L, Han K, Möller E. Clinical significance of isometric bite force versus electrical activity in temporal and masseter muscles. Scand J Dent Res. 1989;97: 539–551. 2617156 10.1111/j.1600-0722.1989.tb00929.x

[29] Dahlberg G. Statistical methods for medical and biological students. New York: Inter Science Publications; 1940.

[30] Aird L, Samuel D, Stokes M. Quadriceps muscle tone, elasticity and stiffness in older males: reliability and symmetry using the MyotonPRO. Arch Gerontol Geriatr. 2012;55: e31–39. 10.1016/j.archger.2012.03.005 22503549

[31] Chuang L, Wu C, Lin K. Reliability, validity, and responsiveness of myotonometric measurement of muscle tone, elasticity, and stiffness in patients with stroke. Arch Phys Med Rehabil. 2012;93: 532–540. 10.1016/j.apmr.2011.09.014 22222143

[32] Dietsch AM, Solomon NP, Sharkey LA, Duffy JR, Strand EA, Clark HM. Perceptual and instrumental assessments of orofacial muscle tone in dysarthric and normal speakers. J Rehabil Res Dev. 2014;51: 1127–1142. 10.1682/JRRD.2013.07.0167 25437151

[33] Gavronski G, Veraksits A, Vasar E, Maaroos J. Evaluation of viscoelastic parameters of the skeletal muscles in junior triathletes. Physiol Meas. 2007;28: 625–637. 10.1088/0967-3334/28/6/002 17664617

[34] Lee H-M, Chen J-JJ, Ju M-S, Lin C-CK, Poon PPW. Validation of portable muscle tone measurement device for quantifying velocity-dependent properties in elbow spasticity. J Electromyogr Kinesiol Off J Int Soc Electrophysiol Kinesiol. 2004;14: 577–589. 10.1016/j.jelekin.2004.02.002 15301776

[35] Schneider S, Peipsi A, Stokes M, Knicker A, Abeln V. Feasibility of monitoring muscle health in microgravity environments using Myoton technology. Med Biol Eng Comput. 2015;53: 57–66. 10.1007/s11517-014-1211-5 25331739

[36] Lion A, Spada RS, Bosser G, Gauchard GC, Anello G, Bosco P, et al “Postural first” principle when balance is challenged in elderly people. Int J Neurosci. 2014;124: 558–566. 10.3109/00207454.2013.864288 24205810

[37] Caron O, Gelat T, Rougier P, Blanchi JP. A comparative analysis of the center of gravity and center of pressure trajectory path lengths in standing posture: an estimation of active stiffness. J Appl Biomech. 2000;16: 234–247. 11757569 10.1123/jab.16.3.234

[38] Alfrey CP, Udden MM, Leach-Huntoon C, Driscoll T, Pickett MH. Control of red blood cell mass in spaceflight. J Appl Physiol Bethesda Md 1985. 1996;81: 98–104. 10.1152/jappl.1996.81.1.98 8828651

[39] Linnarsson D, Hughson RL, Fraser KS, Clément G, Karlsson LL, Mulder E, et al Effects of an artificial gravity countermeasure on orthostatic tolerance, blood volumes and aerobic power after short-term bed rest (BR-AG1). J Appl Physiol Bethesda Md 1985. 2015;118: 29–35. 10.1152/japplphysiol.00061.2014 25342708

[40] Taniguchi H, Tsukada T, Ootaki S, Yamada Y, Inoue M. Correspondence between food consistency and suprahyoid muscle activity, tongue pressure, and bolus transit times during the oropharyngeal phase of swallowing. J Appl Physiol Bethesda Md 1985. 2008;105: 791–799. 10.1152/japplphysiol.90485.2008 18556429

[41] Pereira LJ, Duarte Gaviao MB, Van Der Bilt A. Influence of oral characteristics and food products on masticatory function. Acta Odontol Scand. 2006;64: 193–201. 10.1080/00016350600703459 16829493

[42] Hichijo N, Kawai N, Mori H, Sano R, Ohnuki Y, Okumura S, et al Effects of the masticatory demand on the rat mandibular development. J Oral Rehabil. 2014;41: 581–587. 10.1111/joor.12171 24702545

[43] Kawai N, Sano R, Korfage JAM, Nakamura S, Kinouchi N, Kawakami E, et al Adaptation of rat jaw muscle fibers in postnatal development with a different food consistency: an immunohistochemical and electromyographic study. J Anat. 2010;216: 717–723. 10.1111/j.1469-7580.2010.01235.x 20579175 PMC2952384

[44] Varga S, Spalj S, Lapter Varga M, Anic Milosevic S, Mestrovic S, Slaj M. Maximum voluntary molar bite force in subjects with normal occlusion. Eur J Orthod. 2011;33: 427–433. 10.1093/ejo/cjq097 21062965

[45] Custodio W, Gomes SGF, Faot F, Garcia RCMR, Del Bel Cury AA. Occlusal force, electromyographic activity of masticatory muscles and mandibular flexure of subjects with different facial types. J Appl Oral Sci Rev FOB. 2011;19: 343–349. 10.1590/S1678-77572011005000008 PMC4223785 21655772

[46] Fernandes CP, Glantz POJ, Svensson SA, Bergmark A. A novel sensor for bite force determinations. Dent Mater Off Publ Acad Dent Mater. 2003;19: 118–126. 10.1016/s0109-5641(02)00020-9 12543117

[47] Ferrario VF, Sforza C, Serrao G, Dellavia C, Tartaglia GM. Single tooth bite forces in healthy young adults. J Oral Rehabil. 2004;31: 18–22. 15125591 10.1046/j.0305-182x.2003.01179.x

[48] Viguier M, Dupui P, Montoya R. Posture analysis on young women before and after 60 days of -6 degrees head down bed rest (Wise 2005). Gait Posture. 2009;29: 188–193. 10.1016/j.gaitpost.2008.08.001 18815039

[49] Mulder E, Linnarsson D, Paloski WH, Rittweger J, Wuyts FL, Zange J, et al Effects of five days of bed rest with and without exercise countermeasure on postural stability and gait. J Musculoskelet Neuronal Interact. 2014;14: 359–366. 25198232

[50] Cuccia AM. Interrelationships between dental occlusion and plantar arch. J Bodyw Mov Ther. 2011;15: 242–250. 10.1016/j.jbmt.2010.10.007 21419367

[51] Clément G, Wood SJ. Rocking or Rolling—Perception of Ambiguous Motion after Returning from Space. Chacron MJ, editor. PLoS ONE. 2014;9: e111107 10.1371/journal.pone.0111107 25354042 PMC4213005

[52] Wood SJ, Paloski WH, Clark JB. Assessing Sensorimotor Function Following ISS with Computerized Dynamic Posturography. Aerosp Med Hum Perform. 2015;86: 45–53. 10.3357/AMHP.EC07.2015 26630195

[53] Perinetti G, Türp JC, Primožič J, Di Lenarda R, Contardo L. Associations between the masticatory system and muscle activity of other body districts. A meta-analysis of surface electromyography studies. J Electromyogr Kinesiol Off J Int Soc Electrophysiol Kinesiol. 2011;21: 877–884. 10.1016/j.jelekin.2011.05.014 21802313

[54] Hellmann D, Stein T, Potthast W, Rammelsberg P, Schindler HJ, Ringhof S. The effect of force-controlled biting on human posture control. Hum Mov Sci. 2015;43: 125–137. 10.1016/j.humov.2015.08.009 26282375

[55] Boroojerdi B, Battaglia F, Muellbacher W, Cohen LG. Voluntary teeth clenching facilitates human motor system excitability. Clin Neurophysiol Off J Int Fed Clin Neurophysiol. 2000;111: 988–993. 10.1016/s1388-2457(00)00279-0 10825704

[56] Takada Y, Miyahara T, Tanaka T, Ohyama T, Nakamura Y. Modulation of H reflex of pretibial muscles and reciprocal Ia inhibition of soleus muscle during voluntary teeth clenching in humans. J Neurophysiol. 2000;83: 2063–2070. 10758116 10.1152/jn.2000.83.4.2063

[57] Deriu F, Giaconi E, Rothwell JC, Tolu E. Reflex responses of masseter muscles to sound. Clin Neurophysiol Off J Int Fed Clin Neurophysiol. 2010;121: 1690–1699. 10.1016/j.clinph.2009.11.093 20447862

[58] Tolu E, Pugliatti M. The vestibular system modulates masseter muscle activity. J Vestib Res Equilib Orientat. 1993;3: 163–171. 8275251

[59] Buisseret-Delmas C, Compoint C, Delfini C, Buisseret P. Organisation of reciprocal connections between trigeminal and vestibular nuclei in the rat. J Comp Neurol. 1999;409: 153–168. 10363717 10.1002/(sici)1096-9861(19990621)409:1<153::aid-cne11>3.0.co;2-#

[60] Diagne M, Valla J, Delfini C, Buisseret-Delmas C, Buisseret P. Trigeminovestibular and trigeminospinal pathways in rats: retrograde tracing compared with glutamic acid decarboxylase and glutamate immunohistochemistry. J Comp Neurol. 2006;496: 759–772. 10.1002/cne.20964 16628616

[61] Goto TK, Langenbach GE, Hannam AG. Length changes in the human masseter muscle after jaw movement. Anat Rec. 2001;262: 293–300. 11241197 10.1002/1097-0185(20010301)262:3<293::AID-AR1043>3.0.CO;2-B

[62] Hickenbottom RS, Bishop B, Moriarty TM. Effects of whole-body rotation on masseteric motoneuron excitability. Exp Neurol. 1985;89: 442–453. 3874787 10.1016/0014-4886(85)90103-7

