# Supplementary material for: Craniomandibular System and Postural Balance after 3-Day Dry Immersion
Source: PLoS One. 2016 Feb 25;11(2):e0150052. doi: 10.1371/journal.pone.0150052 (PMC4767814; doi:10.1371/journal.pone.0150052)
Supplement: S1 Table — (PDF) (PDF) [file pone.0150052.s001.pdf]

Supporting Information

S1 Table. Postural sway parameters before, during and after dry immersion. Data (means ± standard error, N = 12) for the two mandibular positions: ICP = dental intercuspidal position, RP = rest position. Days are indicated as follows: control period (BDC-3), the first day of recovery (R0), the second day of recovery period (R+1). **p* < 0.05; ***p* < 0.01 compared with BDC-1 value.

**Parameter**			**Eyes Open**				**Eyes Closed**	
		**BDC-3**	**R0**	**R+1**		**BDC-3**	**R0**	**R+1**
**Path length (mm)**	ICP	212.3 ± 16.7	258.9 ± 19.5*	233.6 ± 16.4		327.7 ± 22.9	392.6 ± 28.5*	347.6 ± 20.9
	RP	200.4 ± 16.8	259.8 ± 22.8**	238.5 ± 14.1**		303.8 ± 26.8	447.9 ± 42.2**	350.0 ± 16.5
**Area (mm^2)**	ICP	47.3 ± 8.1	65.5 ± 18.7	58.0 ± 13.6		99.2 ± 15.0	76.3 ± 11.6	76.8 ± 17.3
	RP	49.0 ± 11.2	76.5 ± 16.9*	47.6 ± 5.0		66.5 ± 7.7	124.3 ± 28.1	72.2 ± 12.1
**Velocity (mm/sec)**	ICP	7.1 ± 0.6	8.6 ± 0.7*	7.8 ± 0.5		10.9 ± 0.8	13.1 ± 1.0*	11.6 ± 0.7
	RP	6.7 ± 0.6	8.7 ± 0.8**	8.0 ± 0.5**		10.1 ± 0.9	14.9 ± 1.4*	11.7 ± 0.6*
**LFS**	ICP	0.6 ± 0.04	0.7 ± 0.06*	0.6 ± 0.05*		0.9 ± 0.07	1.1 ± 0.08	0.9 ± 0.06
	RP	0.5 ± 0.04	0.7 ± 0.07*	0.6 ± 0.04*		0.8 ± 0.08	1.3 ± 0.15*	0.9 ± 0.05
